# Economic Evaluation of Novel Models of Care for Patients With Acute Medical Problems

**DOI:** 10.1001/jamanetworkopen.2023.34936

**Published:** 2023-09-22

**Authors:** Orlanda Q. M. Goh, Xiaohui Xin, Wan Tin Lim, Michelle W. J. Tan, Juliana Y. L. Kan, Hartini Bte Osman, Wanyi Kee, Tse Yean Teo, Wee Boon Tan, Mei Ling Kang, Nicholas Graves

**Affiliations:** 1 Department of Internal Medicine, Singapore General Hospital, Singapore; 2 Medicine Academic Clinical Programme, SingHealth Duke-NUS, Singapore; 3 Health Services Research Unit, Singapore General Hospital, Singapore; 4 SingHealth Duke-NUS Global Health Institute, Singapore; 5 Department of Family Medicine and Continuing Care, Singapore General Hospital, Singapore; 6 Department of Nursing Administration, Singapore General Hospital, Singapore; 7 Population Health and Integrated Care Office, Singapore General Hospital; 8 Department of Infectious Diseases, Singapore General Hospital, Singapore; 9 Programme in Health Services and Systems Research, Duke-NUS Medical School, Singapore

## Abstract

**Importance:**

During COVID-19, Singapore simultaneously experienced a dengue outbreak, and acute hospitals were under pressure to lower bed occupancy rates. This led to new models of care to treat patients with acute, low-severity medical conditions either at home, in a hospital-at-home (HaH) model, or in a clinic-style setting sited at the emergency department in an ambulatory care team (ACT) model, but a reliable cost analysis for these models is lacking.

**Objective:**

To compare personnel costs of HaH and ACT with inpatient care.

**Design, Setting, and Participants:**

In this economic evaluation study, time-driven activity-based costing was used to compare the personnel cost of inpatient care with treating dengue via HaH and treating chest pain via ACT. Participants were patients with nonsevere dengue and chest pain unrelated to a coronary event admitted via the emergency department to the internal medicine service of a tertiary hospital in Singapore.

**Exposures:**

HaH for dengue and ACT for chest pain.

**Main Outcomes and Measures:**

A process map was created for the patient journey for a typical patient with each condition. The amount of time personnel spent on delivering care was estimated and the cost per minute determined based on their wages in 2022. The total cost of care was calculated by multiplying the time spent by the per-minute cost of the personnel resource and summing all costs.

**Results:**

Compared with inpatient care, HaH used 50% less nursing time (418 minutes, 95% uncertainty interval [UI], 370 to 465 minutes) but 80% more medical time (303 minutes, 95% UI, 270 to 338 minutes) per case of dengue. If implemented nationally, HaH would save an estimated 56 828 SGD per year (95% UI, −169 497 to 281 412 SGD [US $41 856; 95% UI, −$124 839 to $207 268]). The probability that HaH is cost saving was 69.2%. Compared with inpatient care, ACT used 15% less nursing time (296 minutes, 95% UI, 257 to 335 minutes) and 50% less medical time (57 minutes, 95% UI, 46 to 69 minutes) per case of chest pain. If implemented nationally, ACT would save an estimated 1 561 185 SGD per year (95% UI, 1 040 666 to 2 086 518 SGD [US $1 149 862; 95% UI, $766 483 to $1 536 786]). The probability that ACT is cost saving was 100%.

**Conclusions and Relevance:**

This economic evaluation found that the HaH and ACT models decreased the overall personnel cost of care. Reorganizing hospital resources may help hospitals reap the benefits of reduced hospital-acquired infections, improved patient recovery, and reduced hospital bed occupancy rates.

## Key Points

**Question:**

What are the personnel costs of novel models of care for acute medical conditions requiring hospital admission?

**Findings:**

In this economic evaluation using time-driven activity-based costing in Singapore, there was evidence that the delivery of care for dengue in hospital at home (HaH) and chest pain in the ambulatory care team (ACT) model can reduce overall costs as compared with usual inpatient services. The probabilities that HaH and ACT are cost-saving were 69.2% and 100%, respectively.

**Meaning:**

With evidence that HaH and ACT can decrease the overall personnel cost of care, it may be worthwhile to reorganize hospital resources to reallocate personnel and set appropriate reimbursement rates for these services.

## Teaser

This economic evaluation uses time-driven activity-based costing to compare the personnel costs of inpatient care vs hospital-at-home for treating dengue and an ambulatory care team model for treating chest pain.

## Introduction

During the COVID-19 pandemic, hospitals were under pressure as patients continued to visit the emergency department (ED) with acute concerns of varying clinical significance and severity.^1^ There was an urgent need to free up beds through implementing new models of care as admitted patients experienced long waiting times in the ED and nonurgent surgeries were cancelled.^2^ Singapore simultaneously faced a dengue outbreak, resulting in added pressure on strained hospital capacity.^3,4^

This led to rapid implementation of novel models of care to treat patients presenting to the ED with acute, low-severity medical conditions such as dengue without hemorrhage and hypotension or chest pain unrelated to a coronary event. Patients with dengue were treated at home, in a hospital-at-home (HaH) model, where patients were admitted to an HaH service from the ED. A multidisciplinary team of physicians and nurses delivered intravenous therapy or perform simple investigations for patients, giving them round-the-clock access to health care personnel from their home.^5^ Patients with chest pain were treated in an ambulatory care team (ACT) model, where timely care was provided in a clinic-style setting at the ED. This model gives patients access to senior physicians and essential investigations and treatment without adding to admission pressures during high hospital bed occupancy.^6^

While emerging from the COVID-19 pandemic, the continued implementation of these models now depends on the cost of running these services. These new models not only free up scarce and expensive hospital capacity, but they are also likely to reduce hospital-acquired infections, length of stay, and overall health care expenditure.^7,8,9,10^ HaH is in line with trends for digitalizing health care and increasing patient and caregiver preferences for recovering at home.^11^ However, a reliable cost analysis of these models is lacking.^5^

In this economic evaluation, we aimed to use time-driven activity-based costing (TDABC) to compare the personnel cost of HaH and ACT models with conventional hospital inpatient care for acute, low-severity medical conditions. TDABC originated in business settings to determine ways in which processes can be redesigned for improvement by showing labor costs at a granular level and assigning them more accurately to service outputs. Stakeholders might understand opportunity costs better and reallocate resources in the improvement of processes.^12,13^ TDABC has been adopted in recent years to health care costing.^14^ It has been applied to various medical conditions and settings by describing a “typical” patient and includes costs related to time spent delivering care to a patient.^12,15,16,17,18,19^ TDABC shows who (personnel completing the task) does what (specific activities performed) when (timing) and for how long (duration of activity).^20^

We hypothesize that the economic advantage in HaH and ACT is the reduction of person-hours required for safe and effective clinical care outside conventional clinical settings. The unknown personnel cost of these services represents an important knowledge gap because this is expected to be the biggest driver of cost savings and a determinant of their scalability and sustainability, especially amid a global shortage of health care personnel.^21,22,23^ Hence, we applied TDABC to compare the personnel cost of conventional hospital inpatient care with the cost of the HaH and ACT models.

## Methods

This cost-minimization analysis followed the Consolidated Health Economic Evaluation Reporting Standards (CHEERS) statement for economic evaluations. The study protocol was submitted to the institutional ethical committee, the Centralized Institutional Review Board, and granted exemption from formal review (reference 2022/2516).

### Selected Conditions and Novel Models of Care

An internal audit of diagnoses of patients admitted to the Department of Internal Medicine was done to select common conditions of low severity and a predictable course of resolution. Chest pain unrelated to an acute coronary event was one of the most frequent admitting diagnoses and was suitable for the ACT model. Patients with this condition had a low risk for deterioration and met the criteria of needing same-day emergency care such as time-sensitive diagnostic tests, intravenous treatment, or procedures over a 12- to 23-hour window, with an early discharge and clinic review. They were managed in the ED to ensure rapid access to medical teams and relevant diagnostics with minimal transfers within the hospital.

Dengue fever with thrombocytopenia but without hemorrhage or hypotension was also a common diagnosis and chosen for the HaH model because monitoring, hydration, and serial blood investigations were required over a few days with a predictable course of recovery. Patients were excluded if they stayed in a home that was beyond a 20-minute drive from the hospital or if there was no caregiver for basic and instrumental activities of daily living if required. Eligible patients with dengue were recruited and discharged home from the ED. On arriving home, they reported vital signs to the team using loaned medical equipment, had blood samples drawn and received intravenous fluid therapy as required, and had round-the-clock access to physicians and nurses. Each morning, they were reviewed physically by nurses and either physically or virtually by physicians depending on their medical condition.

### Time-Driven Activity-Based Costing

In accordance with existing practice for TDABC,^12,14,17,18^ the following tasks were undertaken to compare the personnel cost for HaH and ACT with that for inpatient care for dengue and chest pain, respectively:

1. Creation of a process map for the journey of care for a typical patient with each of the conditions in the specified setting. These were created in consultation with experienced clinicians, nurses, and allied health professionals. It shows each step of services patients will encounter in the health care setting.
2. Estimation of the amount of time that personnel spent on each step in the journey of care. We obtained time estimates from interviewing a range of experienced clinicians, residents, nurses, allied health professionals, administrative staff, and ancillary health care staff. We confirmed these times by shadowing patients and the actual care processes.
3. Calculation of the cost per minute of time for the relevant personnel. We consulted the hospital’s human resource department to determine wage-per-minute estimates based on annualized wages, taking into account individual benefits, bonuses, and the annual wage supplement in 2022.
4. Calculation of the total cost of providing care. We multiplied the time spent delivering care by the per-minute cost of the personnel resource and summed all personnel costs.

### Statistical Analysis

Total personnel costs for each condition in each setting were presented and stratified by designation (physician, nurse, allied health professional, ancillary care staff, or administrative staff) and then by activity type (direct patient care, electronic health record [EHR] documentation, medical handovers, patient communication, and commute). We estimated the differences in cost per year in the hospital by multiplying the cost difference between the 2 models of care per condition by the mean number of patient attendances for that condition per year based on hospital audit data between 2019 and 2021. National estimates for differences in cost per year were derived by scaling up the cost savings in our hospital according to the number of acute hospital beds Singapore General Hospital has (1785) in relation to the total number of acute hospital beds in public hospitals in Singapore (9762).^24^ In Singapore, more than 80% of hospital admissions are to public hospitals.^24^

To account for uncertainty in the estimates, we determined a range of time spent performing each task from the minimum and maximum estimates of time taken. We assumed a uniform distribution of time spent performing a task and obtained 1000 random resamples from this range. Estimates of time spent and corresponding savings are presented as a mean of 1000 resamples with a 95% uncertainty interval (UI). The costing was conducted from the perspective of the health care system because this was the most relevant perspective that would influence the scalability and sustainability of these novel models of care. All costs were reported in 2022 Singapore dollars. The time horizon was the duration of treatment for the episode of care; no discounting for costs was considered for this study as the acute medical conditions studied are treated in a single episode of care and are not expected to require medical follow-up beyond a year.

## Results

**Figure 1.**
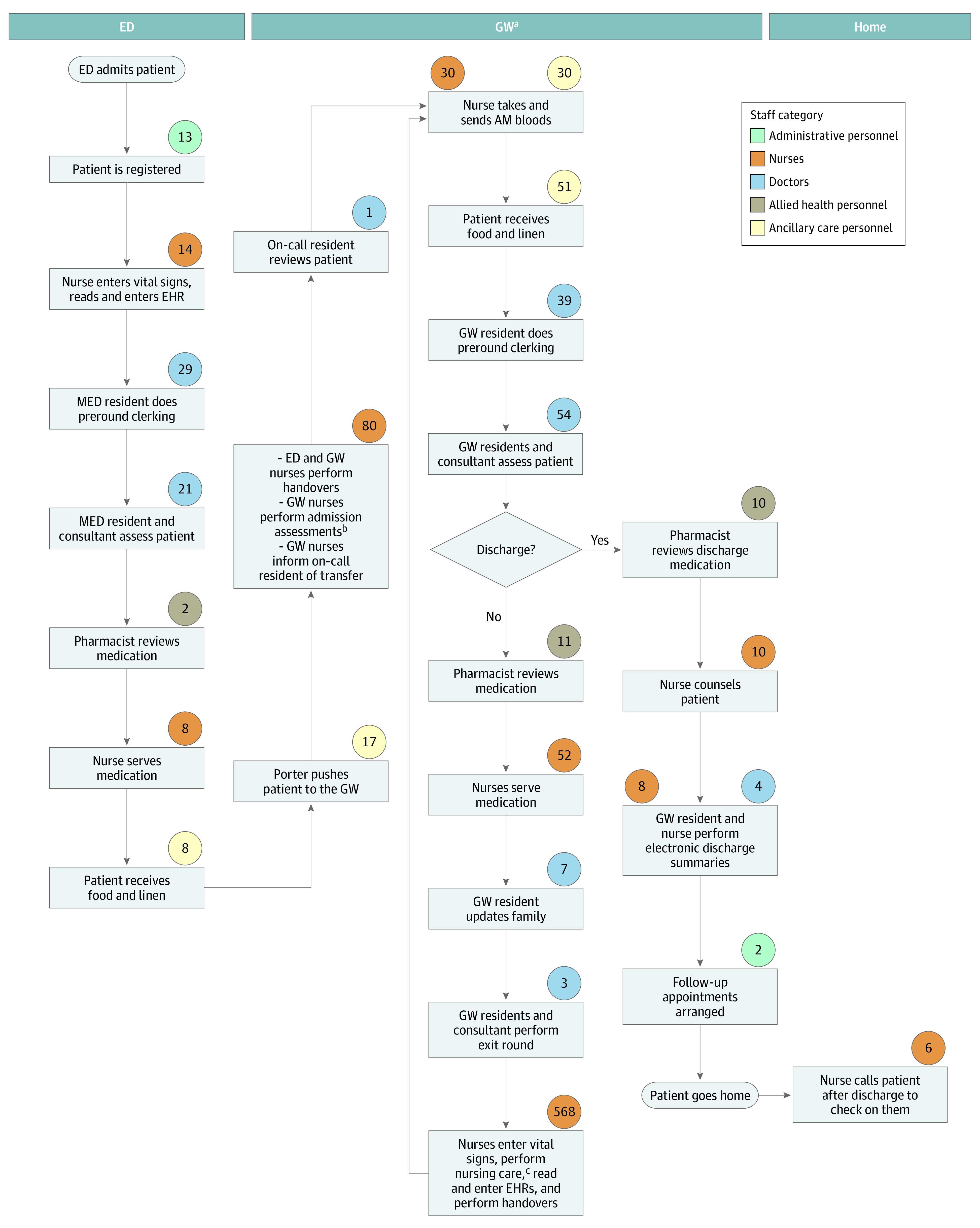
Dengue Treatment in the Hospital Values in circles indicate time in minutes spent on the activity by staff in the related category. AM indicates morning; ED, emergency department; EHR, electronic health record; GW, general ward; MED, medical team in the emergency department. ^a^Based on length of stay of 4 days. ^b^Includes evaluation for skin integrity, vascular examination, swabs to detect carbapenem-resistant *Enterobacterales* and methicillin-resistant *Staphylococcus aureus*, and a nursing care record assessment. ^c^Includes checking intravenous plug sites, providing mouth gargles, assisting in activities of daily living, serving meals, and changing linen.

**Figure 2.**
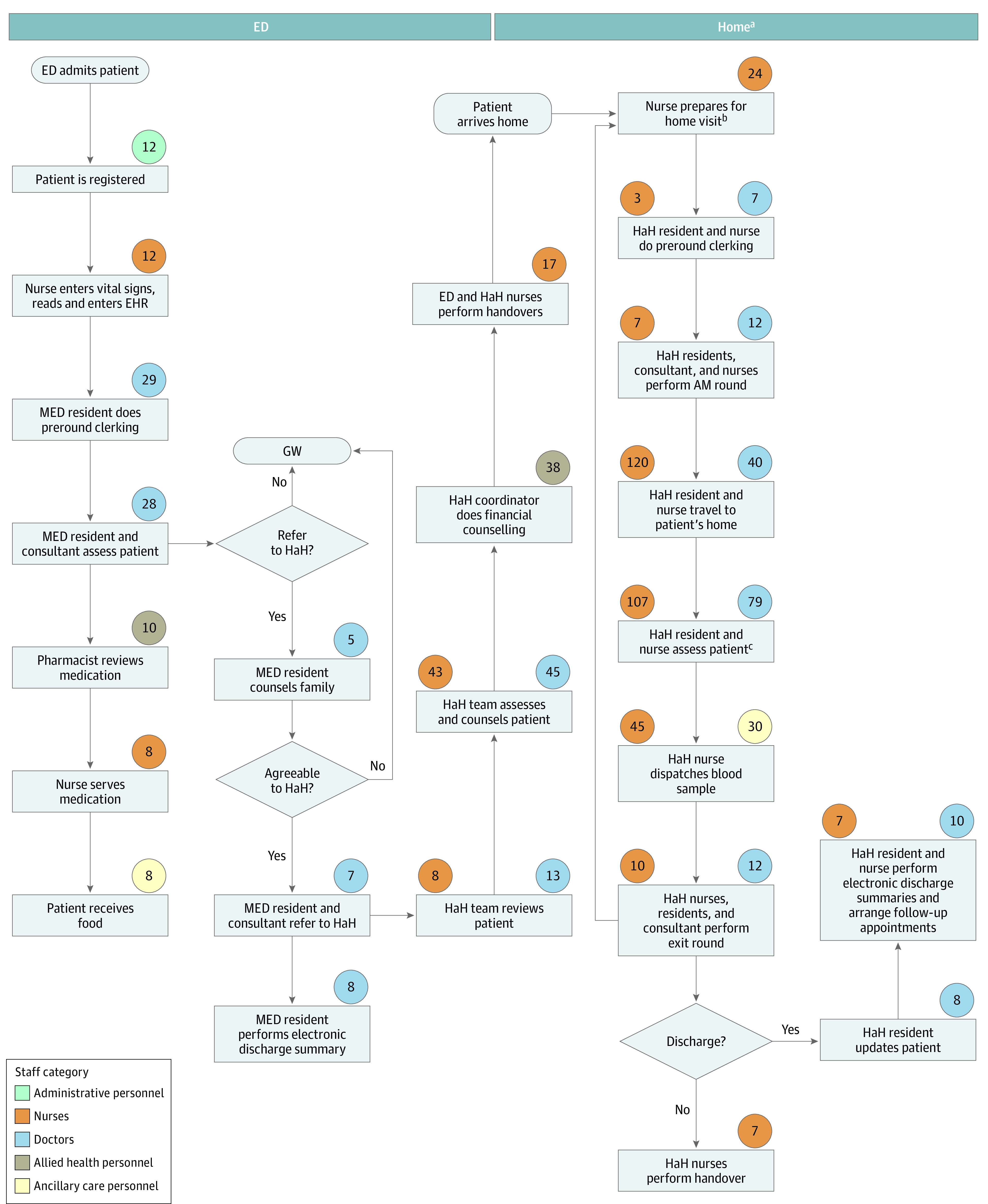
Dengue Treatment via Hospital at Home (HaH) Values in circles indicate time in minutes spent on the activity by staff in the related category. AM indicates morning; ED, emergency department; EHR, electronic health record; GW, general ward; MED, medical team in the emergency department. ^a^Based on length of stay of 4 days. ^b^Includes stock taking and packing medical kit for the home visit and calling the patient to arrange a time for the home visit. ^c^Includes history taking and examination of patient, blood draws, and intravenous infusion as necessary.

**Figure 3.**
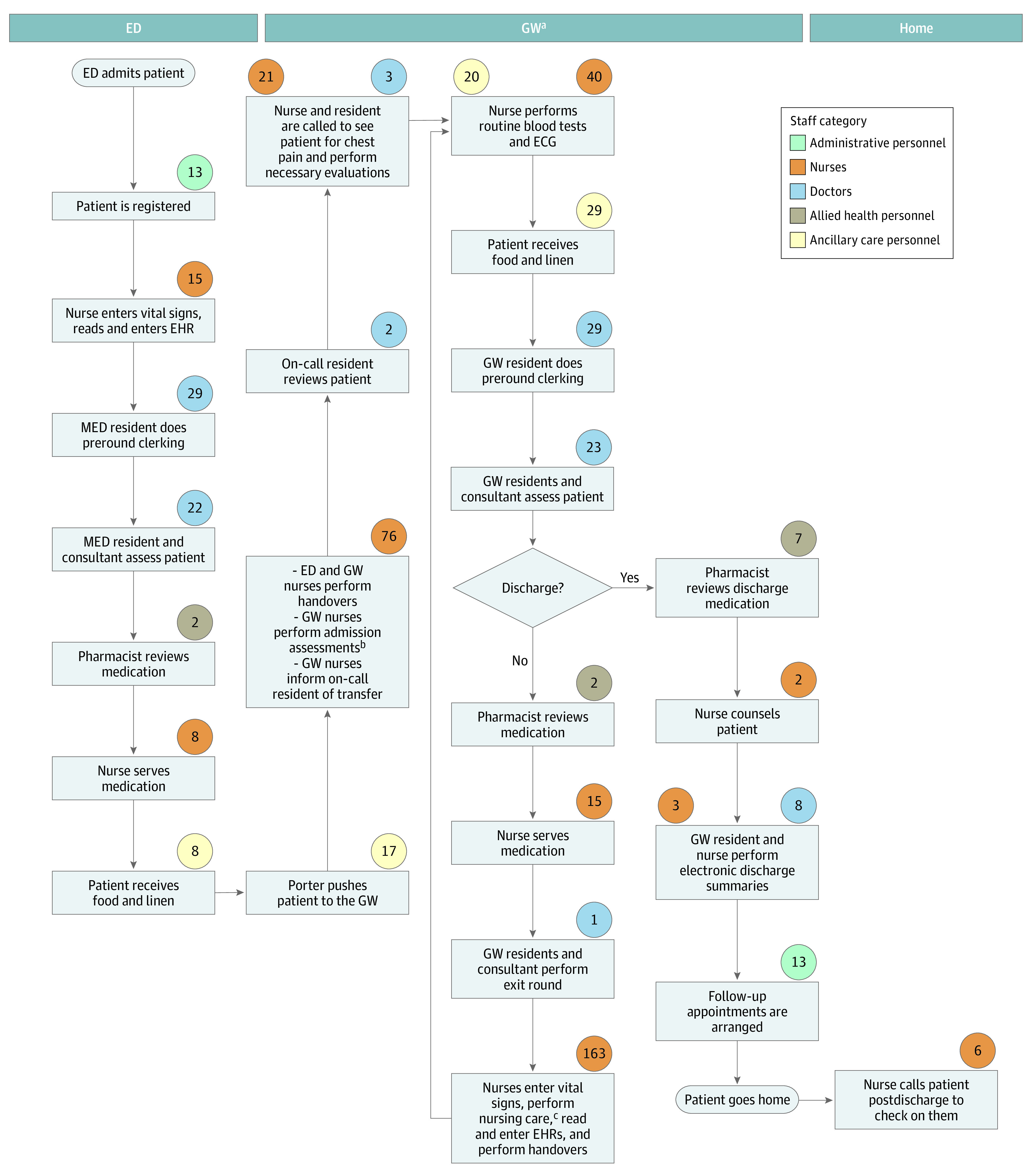
Chest Pain Treatment in the Hospital Values in circles indicate time in minutes spent on the activity by staff in the related category. ECG indicates electrocardiogram; ED, emergency department; EHR, electronic health record; GW, general ward; MED, medical team in the emergency department. ^a^Based on length of stay of 2 days. ^b^Includes evaluation for skin integrity, vascular examination, swabs to detect carbapenem-resistant *Enterobacterales* and methicillin-resistant *Staphylococcus aureus*, and a nursing care record assessment. ^c^Includes checking intravenous plug sites, assisting in activities of daily living, serving meals, and changing linen.

**Figure 4.**
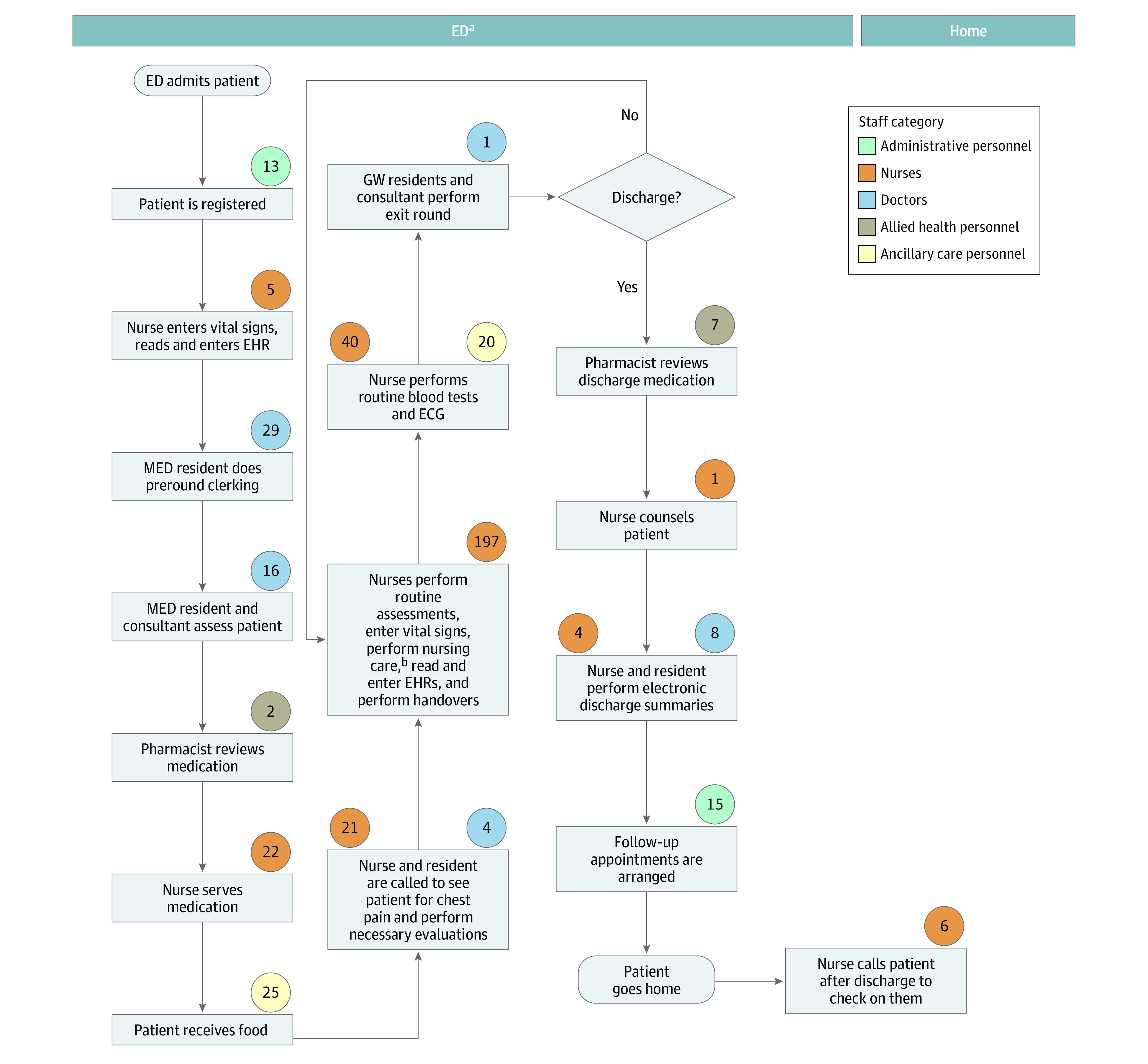
Chest Pain Treatment via the Ambulatory Care Team (ACT) Model Values in circles indicate time in minutes spent on the activity by staff in the related category. ECG indicates electrocardiogram; ED, emergency department; EHR, electronic health record; GW, general ward; MED, medical team in the emergency department. ^a^Based on length of stay of 23 hours. ^b^Includes checking intravenous plug sites.

The process maps for a patient with dengue are shown in Figure 1 and Figure 2. Process maps for chest pain are shown in Figure 3 and Figure 4.

Patients admitted to an inpatient ward in conventional care are transported to a ward located a 4- to 30-minute walk away from the ED by a porter. They are reviewed by multiple medical teams consisting of up to 6 physicians up to twice daily because of the necessary handover of care when a patient’s location changes. These patients also receive thorough nursing assessments, ward orientation, regular nursing care, meals, and linen services.

Patients admitted to HaH are sent home from the ED to receive bed rest and home care advice and are seen via home visits or tele-consultation once a day. They are taught to report their own vital signs and fluid intake to the care team 3 times per day. Nurses draw blood samples and administer intravenous hydration on site as necessary. Patients in the ACT model have a simpler journey because they remain in the ED and are reviewed by a single team of 2 to 3 physicians, receive only essential nursing assessment and care, and are discharged earlier without a transfer of location and health care teams.

Tabulation of the time spent by personnel on each episode of care in the patient’s journey demonstrated many differences in the personnel time spent and cost of care in different models (eTables 1-4 in Supplement 1). In the hospital, 1085 minutes (95% UI, 1018-1151 minutes) or 18 hours was spent on 1 case of dengue (Table). The HaH model used three-quarters of the time (819 minutes; 95% UI, 757-882 minutes). Approximately 50% less nursing time (418 minutes; 95% UI, 370-465 minutes) was needed for HaH. However, HaH used 80% more medical time (303 minutes; 95% UI, 270-338 minutes).

**Table.** Personnel Time Spent and Cost of Delivering Care for Dengue and Chest Pain^a^

Personnel	Time spent, min					Cost, SGD^b^				
	Inpatient		HaH or ACT		Difference^c^	Inpatient		HaH or ACT		Difference^c^
	Mean (95% UI)	%^d^	Mean (95% UI)	%	Mean (95% UI)	Mean (95% UI)	%	Mean (95% UI)	%	Mean (95% UI)
**Dengue (HaH)**										
Medical	165 (147 to 183)	15.2	303 (270 to 338)	37.0	−138 (−178 to −98)	248 (219 to 272)	33.1	401 (359 to 446)	54.9	−154 (−208 to −102)
Nursing	776 (713 to 840)	71.5	418 (370 to 465)	51.1	358 (282 to 430)	438 (403 to 474)	58.5	274 (242 to 305)	37.5	164 (116 to 209)
Allied health	53 (47 to 59)	4.9	77 (54 to 100)	9.4	−24 (−47 to 0)	29 (25 to 33)	3.9	46 (30 to 63)	6.4	−17 (−34 to 0)
Ancillary	76 (63 to 88)	7.0	8 (8 to 8)	1.0	68 (55 to 80)	26 (22 to 31)	3.5	3 (3 to 3)	0.4	24 (19 to 28)
Administrative	15 (6 to 24)	1.4	12 (5 to 20)	1.5	3 (−10 to 16)	7 (3 to 12)	1.0	6 (2 to 10)	0.8	1 (−5 to 8)
Total	1085 (1018 to 1151)	100	819 (757 to 882)	100	266 (175 to 355)	749 (704 to 793)	100	731 (672 to 790)	100	18 (−54 to 89)
**Chest pain (ACT)**										
Medical	117 (102 to 133)	20.4	57 (46 to 69)	13.1	60 (41 to 80)	170 (148 to 192)	40.7	81 (65 to 97)	27.8	89 (61 to 114)
Nursing	347 (312 to 384)	60.5	296 (257 to 335)	68.1	51 (−2 to 103)	199 (180 to 221)	47.9	173 (149 to 196)	59.3	27 (−5 to 59)
Allied health	31 (28 to 35)	5.5	29 (26 to 33)	6.8	2 (−3 to 7)	17 (14 to 19)	4.0	15 (13 to 18)	5.2	1 (−2 to 5)
Ancillary	54 (41 to 67)	9.4	25 (25 to 25)	5.7	29 (16 to 42)	19 (14 to 23)	4.5	9 (9 to 9)	3.0	10 (6 to 15)
Administrative	25 (12 to 37)	4.3	28 (20 to 35)	6.3	−3 (−18 to 13)	12 (6 to 18)	2.9	13 (10 to 17)	4.6	−1 (−9 to 6)
Total	574 (532 to 620)	100	435 (391 to 476)	100	139 (79 to 201)	416 (386 to 447)	100.0	291 (260 to 320)	100	126 (84 to 168)

Abbreviations: ACT, ambulatory care team; HaH, hospital-at-home; UI, uncertainty interval.

^a^ Estimates are based on the time spent for a typical case of low severity and rounded to the nearest whole number for time and cost.

^b^ Costs are shown in 2022 Singapore dollars.

^c^ Difference equals inpatient minus HaH for dengue and inpatient minus ACT for chest pain.

^d^ Percentage of all time spent based on mean time spent.

The personnel cost of treating dengue was 749 SGD (95% UI, 704 to 793 SGD [US $552; 95% UI, $519 to $584]) in the hospital, compared with 731 SGD (95% UI, 672 to 790 SGD [US $538; 95% UI, $495 to $582]) in HaH. HaH was estimated to save 18 SGD per case (95% UI, −54 to 89 SGD [US $13; 95% UI, −$40 to $66]) or 10 391 SGD per year (95% UI, −30 993 to 51 457 SGD [US $7653; 95% UI, −$22 827 to $37 900]) based on a mean number of 575 cases of dengue per year. If this program were implemented nationally, personnel cost savings were estimated to be 56 828 SGD (95 UI, −169 497 to 281 412 SGD [US $41 856; 95% UI, −$124 839 to $207 268]). The probability that HaH is cost-saving was 69.2% (eFigure 1 in Supplement 1).

Treating chest pain in the hospital used 574 minutes (95% UI, 532-620 minutes) or 10 hours of personnel time. The ACT model used three-quarters of the time (435 minutes; 95% UI, 391-476 minutes). Fifteen percent less nursing time (296 minutes; 95% UI, 257-335 minutes) and 50% less medical time (57 minutes; 95% UI, 46-69 minutes) were needed in ACT.

The personnel cost of treating chest pain was 416 SGD (95% UI, 386-447 SGD [US $306; 95% UI, $284-$329]) in the hospital, compared with 291 SGD (95% UI, 260-320 SGD [US $214; 95% UI, $192-236]) in the ACT model. ACT was estimated to save 126 SGD per case (95% UI, 84-168 SGD [US $91; 95% UI, $62-$124]) or 285 466 SGD a year (95% UI, 190 288-381 524 SGD [US $210 255; 95% UI, $140 153-$281 005]) based on a mean number of 2274 cases per year. If this program were implemented nationally, personnel cost savings were estimated to be 1 561 185 SGD (95% UI, 1 040 666-2 086 518 SGD [US $1 149 862; 95% UI, $766 483-$1 536 786]). The probability that ACT is cost-saving was 100% (eFigure 2 in Supplement 1).

In the hospital, nursing care and medical care were the main drivers of cost, accounting for 48% to 59% and 33% to 41% of personnel costs, respectively (Table). For physicians, 51% to 57% of their time was spent on EHRs, compared with 31% to 32% of time spent on direct patient interaction or communication (eTable 5 in Supplement 1). In 1 year, managing dengue and chest pain in our hospital was estimated to be associated with 813 and 2519 person-hours of physician time spent on EHRs, respectively.

## Discussion

We provide some evidence that the delivery of care for dengue in HaH and chest pain in the ACT model can reduce overall costs as compared with business-as-usual services. A large proportion of the savings are attributed to a reduced burden on nursing and physician time, as well as eliminating unnecessary “bed and breakfast” services in treating these conditions of low acuity and severity. By design, these alternative models deliver treatment to patients according to existing clinical practice guidelines and are expected to result in the same clinical outcomes in addition to the decreased risk of hospital-acquired infections and improved patient-reported outcomes related to quality of sleep and increased convenience from an earlier return to home.^1,2,3,4,5^ These models that reduce inpatient bed utilization are of greater value in postpandemic Singapore as bed occupancy rates in public hospitals frequently remain above 85%.^25,26^

In the HaH model, there was an increase in medical personnel time spent on all aspects of care. This is due to the extra time spent assessing patients for red flags that preclude safe recovery at home, such as early warning signs of severe dengue and anticipated poor venous access, reviewing EHRs to assess their suitability for HaH based on additional sociodemographic information, and planning logistics around the delivery of medications and laboratory tests and patient transportation outside current protocols, conventions, and office hours. There is also significant time spent counseling patients and families on this service and traveling for home visits. Although there was a large reduction in routine nursing care, nurses spent more time on care coordination. Hence, the HaH model resulted in modest cost savings. Nevertheless, counting the time and cost of medical personnel required for the safe and effective implementation of this novel service has important implications: appropriate reallocation of physicians and nurses to this service and reimbursement are needed to guarantee its scale and sustainability and should be taken into consideration for workforce projections.^27^

In the ACT model, physicians spent less time on all aspects of care for chest pain because of leaner teams and the avoidance of unnecessary transfers of care of a patient between sites on a large hospital campus. This eliminates the number of physicians who review patients with a simple condition and the time needed to retake a history, establish rapport, repeat medical advice that was already given, and create and review additional documents in the EHR. The use of EHRs and computers has been increasingly burdensome to health care professionals in part because of increasing demands on medical documentation by a litigious society while negatively affecting the patient-physician interaction and patients’ perception of their care.^28,29^ Reducing these components of care that do not add value reduces the time burden on a health care service and risk of health care worker burnout, in addition to increasing work productivity.^30,31^ This is increasingly important in the midst of a global shortage of health care workers.^23^

If the HaH model were operated by all acute hospitals nationally, costs would be expected to decrease as commute times decrease when patients are serviced in smaller geographical zones. Increased public familiarity with the incorporation of this service into existing public health financing schemes would decrease the time needed to counsel patients on enrollment.^32^ However, the expansion of this service to other more complex conditions would be dependent on available protocols and resources to facilitate timely return to hospitals for investigation and treatment, as well as patient and clinician acceptance of these potential “U-turns.” Unfortunately, patients who are socially isolated and who cannot recover without a caregiver would always be excluded from this service.

In sparsely populated countries or rural areas with less established health care infrastructure, HaH may be a viable option with emerging digital health monitoring technology, telehealth, and satellite centers that provide basic investigations and dispense medications. In low-resource settings, task-shifting these elements of service delivery to community health workers increases affordable access to care.^33^ Both HaH and ACT models increase the efficiency of health care delivery while using less hospital real estate for simple acute conditions and are therefore important options for both high- and low-resource countries that face the universal problem of rising costs of construction.

We suggest that the strength of our study lies in its importance in informing hospitals and health financing decision-making.^34^ In the implementation of new services without set reimbursement rates and with variation among various clinicians, micro-costing is needed to understand relative costs with precision especially for personnel costs, which are an important driver of cost in medical care.^16^ By describing the personnel cost of new services in terms of actual personnel utilization, we show what inputs are needed to conscientiously reorganize hospital resources and services to ensure safe and quality care for patients under the HaH and ACT models. This makes a case for updating current methods of reimbursement to health care workers to appropriately compensate the reallocation of personnel resources to provide round-the-clock care in these high-value cost-saving settings that exceed the traditional boundaries of care.

Our study also underscores the need for systematic collection of data on care processes and its integration with clinical and economic data to inform the evaluation of health care delivery models.^35^ We illustrate how this can be done by adopting a pragmatic approach for describing care processes as modular units, such as time spent taking vital parameters or performing venipuncture, so that this can be generalizable to other conditions and settings. By categorizing cost by personnel activity and type, we have highlighted strategies to reduce the burden of care amid rising health care costs and a global shortage of health care workers.

### Limitations

Our study has some limitations. We were able to compare processes and cost only in simple conditions with predictable pathways and outcomes. Chronic comorbid conditions with associated social risk factors are more variable in management, and outcome cannot be modeled and compared consistently. These HaH and ACT models do not take into account the time needed to engage students and residents to teach during ward rounds, which has high value to the medical ecosystem but not within the scope of this study.

## Conclusions

In this economic evaluation, we found that innovative health care services such as the HaH and ACT models decreased the overall personnel cost of care. To reap the benefits of reduced hospital-acquired infections, improved patient recovery, and reduced hospital bed occupancy rates, it may be worthwhile to reorganize hospital resources to efficiently allocate personnel and set appropriate reimbursement rates for these services.

## Notes

Supplement 1. **eTable 1.** Dengue cost tabulation, inpatient model with length of stay of 4 days **eTable 2.** Dengue cost tabulation, HaH model with length of stay of 4 days **eTable 3.** Chest pain cost tabulation, inpatient model with length of stay of 2 days **eTable 4.** Chest pain cost tabulation, ACT model with length of stay of 23 hours **eFigure 1.** Distribution of national annual cost savings for HaH model of care for dengue **eFigure 2.** Distribution of national annual cost savings for ACT model of care for chest pain **eTable 5.** Breakdown of activity type for care delivered to patients by physicians

Supplement 2.
Data Sharing Statement
