# Supplementary material for: Economic Evaluation of Novel Models of Care for Patients With Acute Medical Problems
Source: JAMA Netw Open. 2023 Sep 22;6(9):e2334936. doi: 10.1001/jamanetworkopen.2023.34936 (PMC10517377; doi:10.1001/jamanetworkopen.2023.34936)
Supplement: Supplement 1. — eTable 1. Dengue cost tabulation, inpatient model with length of stay of 4 days eTable 2. Dengue cost tabulation, HaH model with length of stay of 4 days eTable 3. Chest pain cost tabulation, inpatient model with length of stay of 2 days eTable 4. Chest pain cost tabulation, ACT model with length of stay of 23 hours eFigure 1. Distribution of national annual cost savings for HaH model of care for dengue eFigure 2. Distribution of national annual cost savings for ACT model of care for chest pain eTable 5. Breakdown of activity type for care delivered to patients by physicians [file jamanetwopen-e2334936-s001.pdf]

Supplemental Online Content

Goh OQM, Xin X, Lim WT, et al. Economic evaluation of novel models of care for patients with acute medical problems. *JAMA Netw Open*. 2023;6(9):e2334936. doi:10.1001/jamanetworkopen.2023.34936

eTable 1. Dengue cost tabulation, inpatient model with length of stay of 4 days
eTable 2. Dengue cost tabulation, HaH model with length of stay of 4 days
eTable 3. Chest pain cost tabulation, inpatient model with length of stay of 2 days
eTable 4. Chest pain cost tabulation, ACT model with length of stay of 23 hours
eFigure 1. Distribution of national annual cost savings for HaH model of care for dengue
eFigure 2. Distribution of national annual cost savings for ACT model of care for chest pain
eTable 5. Breakdown of activity type for care delivered to patients by physicians

This supplemental material has been provided by the authors to give readers additional information about their work.

© 2023 Goh OQM et al. *JAMA Network Open.*

eTable 1. Dengue cost tabulation, inpatient model with length of stay of 4 days

Staff Category	Job Category	Time in ED	Time in GW	Total time	Total time per category	Minute wage	ED cost	GW cost	Personnel cost	Total personnel cost per category
Administrative	Associate executive and other support staff	8	3	11	11	0.49	3.92	1.47	5.39	5.39
Allied Health	Laboratory assistant and other AH support staff	0	30	30		0.42	0	12.6	12.6	
Allied Health	Pharmacist, research coordinator and other AHP	2	23	25	55	0.72	1.44	16.56	18	30.6
Ancillary	Chef, PCA	25	51	76	76	0.35	8.75	17.85	26.6	26.6
Nursing	Enrolled nurse	38	291	329		0.44	16.72	128.04	144.76	
Nursing	Staff nurse	17	459	476	805	0.66	11.22	302.94	314.16	458.92
Medical	Resident	41	97	138		1.12	45.92	108.64	154.56	
Medical	Consultant	8	18	26	164	3.15	25.2	56.7	81.9	236.46
TOTAL		139	972	1111	1111	0	113.17	644.8	757.97	757.97

*Time and cost values are shown as an output of one sample simulation and are thus numerically different from the estimates of mean and 95% uncertainty interval reported in manuscript Table 1.
AH: Allied health
AHP: Allied health professional
PCA: Patient care assistant

© 2023 Goh OQM et al. *JAMA Network Open.*

eTable 2. Dengue cost tabulation, HaH model with length of stay of 4 days

Staff Category	Job Category	Time in ED	Time in HaH	Total time	Total time per category	Minute wage	ED cost	HaH cost	Personnel cost	Total personnel cost by category
Administrative	Associate executive and other support staff	13	0	13	13	0.49	6.37	0	6.37	6.37
Allied Health	Laboratory assistant and other AH support staff	30	0	30		0.42	12.6	0	12.6	
Allied Health	Pharmacist, research coordinator and other AHP	9	20	29	59	0.72	6.48	14.4	20.88	33.48
Ancillary	Chef, PCA	8	0	8	8	0.35	2.8	0	2.8	2.8
Nursing	Enrolled nurse	9	0	9		0.44	3.96	0	3.96	
Nursing	Staff nurse	15	408	423	432	0.66	9.9	269.28	279.18	283.14
Medical	Resident	75	216	291		1.12	84	241.92	325.92	
Medical	Consultant	15	12	27	318	3.15	47.25	37.8	85.05	410.97
TOTAL		174	656	830	830	0	173.36	563.4	736.76	736.76

*Time and cost values are shown as an output of one sample simulation and are thus numerically different from the estimates of mean and 95% uncertainty interval reported in manuscript Table 1.
AH: Allied health
AHP: Allied health professional
PCA: Patient care assistant

© 2023 Goh OQM et al. *JAMA Network Open.*

eTable 3. Chest pain cost tabulation, inpatient model with length of stay of 2 days

Staff Category	Job Category	Time in ED	Time in GW	Total time	Total time per personnel	Minute wage	ED Cost	GW cost	Personnel cost	Total personnel cost per category
Administrative	Associate executive and other support staff	19	14	33	33	0.49	9.31	6.86	16.17	16.17
Allied Health	Laboratory assistant and other AH support staff	0	20	20		0.42	0	8.4	8.4	
Allied Health	Pharmacist, research coordinator and other AHP	1	7	8	28	0.72	0.72	5.04	5.76	14.16
Ancillary	Chef, PCA	37	29	66	66	0.35	12.95	10.15	23.1	23.1
Nursing	Enrolled nurse	23	100	123		0.44	10.12	44	54.12	
Nursing	Staff nurse	17	207	224	347	0.66	11.22	136.62	147.84	201.96
Medical	Resident	36	54	90		1.12	40.32	60.48	100.8	
Medical	Consultant	10	7	17	107	3.15	31.5	22.05	53.55	154.35
TOTAL		143	438	581	581	0	116.14	293.6	409.74	409.74

*Time and cost values are shown as an output of one sample simulation and are thus numerically different from the estimates of mean and 95% uncertainty interval reported in manuscript Table 1.
AH: Allied health
AHP: Allied health professional
PCA: Patient care assistant

© 2023 Goh OQM et al. *JAMA Network Open.*

eTable 4. Chest pain cost tabulation, ACT model with length of stay of 23 hours

Staff Category	Job Category	Time in ACT	Total time per personnel	Minute wage	Personnel cost	Total personnel cost by category
Administrative	Associate executive and other support staff	32	32	0.49	15.68	15.68
Allied Health	Laboratory assistant and other AH support staff	20		0.42	8.4	
Allied Health	Pharmacist, research coordinator and other AHP	13	33	0.72	9.36	17.76
Ancillary	Chef, PCA	25	25	0.35	8.75	8.75
Nursing	Enrolled nurse	106		0.44	46.64	
Nursing	Staff nurse	205	311	0.66	135.3	181.94
Medical	Resident	51		1.12	57.12	
Medical	Consultant	7	58	3.15	22.05	79.17
TOTAL	0	459	459	0	303.3	303.3

*Time and cost values are shown as an output of one sample simulation and are thus numerically different from the estimates of mean and 95% uncertainty interval reported in manuscript Table 1.
AH: Allied health
AHP: Allied health professional
PCA: Patient care assistant

© 2023 Goh OQM et al. *JAMA Network Open.*

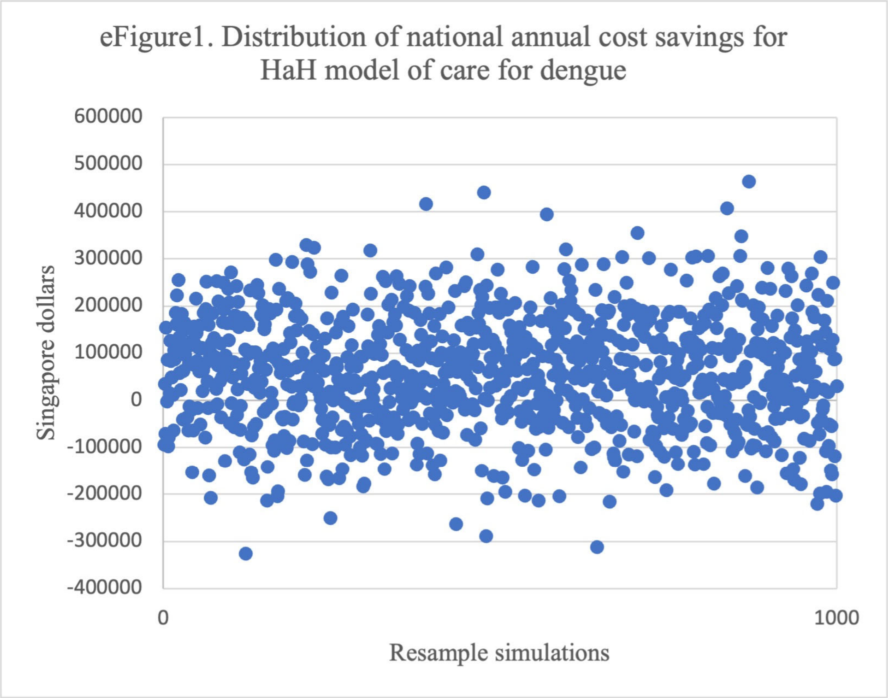

eFigure1. Distribution of national annual cost savings for HaH model of care for dengue

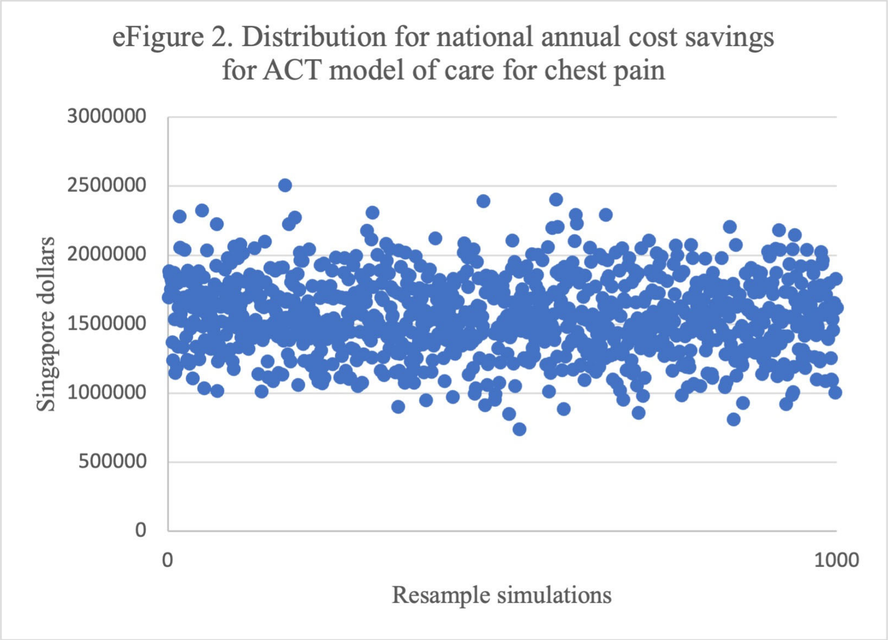

eFigure 2. Distribution for national annual cost savings for ACT model of care for chest pain

© 2023 Goh OQM et al. *JAMA Network Open.*

eTable 5. Breakdown of activity type for care delivered to patients by physicians

**Personnel**	**Time spent, minutes**				
Dengue					
	Inpatient		HaH		Difference*
	Mean (95% UI#)	%^	Mean (95% UI)	%	Mean (95% UI)
Direct patient care	22 (19,25)	13.3	63 (48,77)	20.8	-41 (-56,-26)
Electronic health records	85 (73,97)	51.3	99 (84,114)	32.6	-14 (-32,6)
Medical communication	28 (23,32)	16.6	36 (30,42)	11.9	-9 (-16,0)
Patient communication	31 (18,43)	18.8	65 (52,79)	21.4	-34 (-53,-15)
Commute	0 (0,0)	0.0	40 (21,59)	13.3	-40 (-59,-21)
Total	165 (147,183)	100.0	303 (270,338)	100.0	-138 (-178,-98)
Chest pain					
	Inpatient		ACT		Difference*
	Mean (95% UI#)	%^	Mean (95% UI)	%	Mean (95% UI)
Direct patient care	20 (15,23)	16.7	9 (6,12)	15.8	11 (5,15)
Electronic health records	66 (55,78)	56.9	33 (25,41)	57.9	34 (19,47)
Medical communication	14 (11,16)	12.0	6 (4,8)	10.6	8 (5,11)
Patient communication	17 (8,26)	14.4	9 (2,15)	15.7	8 (-4,20)
Total	117 (102,133)	100.0	57 (46,69)	100.0	60 (41,80)

UI: Uncertainty interval

^ Percentage of all time spent based on mean time spent

* Difference = Inpatient – HAH for dengue; Inpatient – ACT for chest pain

© 2023 Goh OQM et al. *JAMA Network Open.*
