# Supplementary material for: Economic Evaluation of Novel Models of Care for Patients With Acute Medical Problems
Source: JAMA Netw Open. 2023 Sep 22;6(9):e2334936. doi: 10.1001/jamanetworkopen.2023.34936 (PMC10517377; doi:10.1001/jamanetworkopen.2023.34936)
Supplement: Supplement 2. — Data Sharing Statement [file jamanetwopen-e2334936-s002.pdf]

Data Sharing Statement

Goh. Economic Evaluation of Novel Models of Care for Patients With Acute Medical Problems. *JAMA Netw Open*. Published September 21, 2023. doi:10.1001/jamanetworkopen.2023.34936

Data
Data available: No

Additional Information
Explanation for why data not available: All available data is already provided in the tables figures and supplements
